# Combination of β-glucan and *Morus alba* L. Leaf Extract Promotes Metabolic Benefits in Mice Fed a High-Fat Diet

**DOI:** 10.3390/nu9101110

**Published:** 2017-10-12

**Authors:** Jie Xu, Xiaojie Wang, Ke Cao, Zhizhong Dong, Zhihui Feng, Jiankang Liu

**Affiliations:** 1 Center for Mitochondrial Biology and Medicine, The Key Laboratory of Biomedical Information Engineering of Ministry of Education, School of Life Science and Technology and Frontier Institute of Science and Technology, Xi’an Jiaotong University, Xi’an 710049, China; ff.89.03.22@stu.xjtu.edu.cn (J.X.); fzh25@163.com (X.W.); caoke@stu.xjtu.edu.cn (K.C.); 2 Nestlé Research Center Beijing, Beijing 100095, China; dongzz@cofco.com

**Keywords:** β-glucan, high-fat diet, metabolic disorders, mulberry, fatty liver

## Abstract

β-glucan (BG) and mulberry have received increasing attention for their benefits as natural sources of metabolic health. In the current study, we investigated the synergetic beneficial effects of BG and mulberry leaf extract (MLE) in mice fed a high-fat diet (HFD). Male C57BL6 mice were fed a HFD for twelve weeks to induce significant obesity and insulin resistance. BG and MLE were administrated orally throughout the feeding period. The administration of BG resulted in a significant reduction in body weight gain, perirenal fat mass, fasting insulin, serum lipids, serum inflammation markers, and fatty liver, showing systemic health improvement. Likewise, the administration of MLE showed benefits similar to BG, with the exception of body weight gain. In addition to the systemic benefits, the combination of BG and MLE resulted in a synergetic improvement in insulin sensitivity. Meanwhile, only the combination of BG and MLE significantly enhanced liver GST (Glutathione S-Transferase) activity and CuZn–SOD (Superoxide dismutase (Cu-Zn)) activity, resulting in a significant reduction in GSH/GSSG (Glutathione disulfide) and reactive oxygen species (ROS) levels in the liver. These results further confirm the beneficial effects of BG and MLE on metabolic disorders and show that the combination of BG and MLE has synergetic effects.

## 1. Introduction

Metabolic syndrome (MS), a clustering of risk factors, including hyperglycemia, hypertension, dyslipidemia, insulin resistance and central adiposity [1], leads to an increased prevalence of cardiovascular diseases (CVD) and type 2 diabetes mellitus (T2DM), which contributes to an enormous loss of quality of life, in both developed and developing nations [2]. A recent study indicated that the mortality for T2DM is approximately five million per year, and it is expected that 23.6 million will die of CVD worldwide by 2030 [3]. As these conditions are among the leading causes of deaths worldwide, preventing metabolic syndrome development is of critical importance [4]. Since a suboptimal lifestyle, which includes excessive caloric intake, an unbalanced diet, chronic stress, and physical inactivity, is said to be the major contributor to MS development [5,6,7], nutritional and physical interventions are still considered effective strategies to improve metabolic health.

β-glucans (BG) are soluble fibers that are located in the endosperm cell walls of cereals that are rich in oat and barley [8]. Among the different naturally occurring substances, BG has gained much interest in the field of functional foods due to its potential health benefits, such as modulating the immune system, anti-infective and anti-tumorigenic activities, etc. [9,10]. More recent studies also suggest that BG is beneficial for improving metabolic disorders. The administration of BG re-establishes the redox balance and activates an antioxidant and anti-inflammatory mechanism in streptozotocin-induced diabetic rats [11,12]. The consumption of barley BG ameliorates fatty liver and insulin resistance in high-fat diet-induced diabetic mice [13]. Although conflicting results have been obtained from human studies, the consumption of BG is still suggested to improve the glycemic index of meals and glucose metabolism in patients with MS, as well as in healthy subjects [14].

Mulberry is another popular food that has a long history in Chinese traditional medicine. Although its fruit is the popular part being consumed, its leaves are also reported to contain various phytochemical constituents, such as flavonoids and polyphenol compounds, which may contribute to the beneficial effects of mulberry leaf extract (MLE) on metabolic disorders [15], and 1-deoxynojirimycin (DNJ) is the crucial and extensively studied cofactor, responsible for helping control blood glucose levels in diabetic patients [16]. MLE was recently shown to improve obesity by inducing adipocyte apoptosis and inhibiting preadipocyte differentiation and hepatic lipogenesis in HFD-induced obese mice [17], and correcting hyperglycemia and increasing insulin secretion by MLE was also demonstrated in a streptozotocin (STZ)-induced non-obese diabetic rat model [18]. Although both BG and MLE have shown potential to improve the MS condition in most animal studies, the relative high administration doses are not suitable for human consumption. Therefore, in the present study, we investigated lower doses of BG and MLE in HFD-induced obese and diabetic mice and explored their potential synergetic benefits on MS.

## 2. Materials and Methods

### 2.1. Materials

DCF-DA (2′,7′-dichlorofluorescein) was purchased from Invitrogen (Carlsbad, CA, USA) and 2,3-naphthalenedicarboxyaldehyde (NDA) was purchased from Sigma (St. Louis, MO, USA). The ELISA (Enzyme-linked immunosorbent assay) kits for insulin, leptin, IL-1β (Interleukin 1beta), IL-4, and TNFα (Tumor necrosis factor alpha) were purchased from RD systems (Shanghai, China). BG and MLE powder were provided by the Nestlé RD center (Beijing, China). Mulberry (*Morus alba* L.) leaves were collected in June, and the extraction was prepared with water and ethanol following a published protocol [19]; the compound 1-Deoxynojirimycin (DNJ) was tested as a quality control (1%). The obtained ethanol extracts of mulberry leaf were kept at −80 °C until further use.

### 2.2. Animals and Treatments 

Four-week-old male C57BL/6 mice were purchased from the SLAC (Shanghai Laboratory Animal Center) Laboratory Animal Co. Ltd. (Shanghai, China) and were housed in a temperature (22–28 °C)- and humidity (60%)-controlled animal room, in a 12 h light/12 h dark cycle (light from 8:00 to 20:00) with food and water provided during the experiments. After 1 week of acclimatization, the mice were randomly divided into five groups (*n* = 10 in each group) as follows: mice fed a normal diet (control, 10% kcal fat content); mice fed a high-fat diet (HFD, 60% kcal fat content); mice fed a high-fat diet with a daily oral gavage of BG (200 mg/kg/day, HFD + BG); mice fed a high-fat diet with a daily oral gavage of MLE (200 mg/kg/day, HFD + MLE); and mice fed a high-fat diet with a daily oral gavage of both BG and MLE (HFD + BG + MLE). Both BG and MLE were prepared in water for gavage. After 12 weeks, the mice were fasted overnight and sacrificed. The protocol of the present study was approved by the Animal Care and Use Committee of School of Life Science and Technology, Xi’an Jiaotong University (2015-0016). All of the procedures were performed in accordance with the United States Public Health Services Guide for the Care and Use of Laboratory Animals, and all possible efforts were made to minimize the suffering and number of animals utilized in this study.

### 2.3. Oral Glucose Tolerance Test (OGTT) 

An OGTT was performed after 12 weeks of feeding and gavage. All mice were fasted overnight before the test. Blood was taken from the retrobulbar vein, both before and 15, 30, 60, and 120 min after a glucose gavage (1 g/kg body weight). The plasma glucose concentration was determined using the glucose oxidation method.

### 2.4. Tissue and Serum Samples Preparation

After the mice were sacrificed, the liver and visceral fat pads (including the perirenal and epidydimal fat pads) were removed, weighed and stored at −80 °C until further analysis. Blood samples were obtained by cardiac puncture, and serum was separated by centrifugation (3000 rpm for 10 min). The triglyceride (TG), total cholesterol (TC), LDL (low-density lipoprotein) cholesterol, ALT (Alanine Aminotransferase), and AST (Aspartate Aminotransferase) levels were analyzed with an automated biochemistry analyzer (Hitachi Ltd., Tokyo, Japan). The serum levels of TNFα, free fatty acids, insulin, leptin, IL-1β, and IL-6 were measured using commercial kits, according to the manufacturers’ standards and protocols (R & D Systems, Shanghai, China).

### 2.5. Histological Analysis of the Liver Samples

The liver tissue was excised, washed with ice-cold phosphate-buffered saline (PBS) and placed in 10% formalin. Several sections of tissue (thickness of 4–5 μm) were prepared and stained with hematoxylin and eosin (H and E) for histopathology and were visualized by an Olympus BX71 microscope (Hachioji, Japan).

### 2.6. Analysis of Hepatic Lipids 

The liver tissues were collected and homogenized in ice-cold PBS after centrifugation (1000× *g*, 10 min), and the supernatant was collected for analysis. The concentrations of the liver triglyceride and total cholesterol were analyzed using commercial clinical diagnosis kits according to the manufacturers’ protocols (Jiancheng, Jiangsu, China). 

### 2.7. Biochemical Analysis 

Small portions of the liver tissue and muscle tissue were collected and homogenized in ice-cold phosphate buffered saline (PBS). After centrifugation (1000× *g*, 10 min), the supernatant was collected for analysis. Glutathione (GSH) and GSSG levels, as well as GST and CuZn–SOD activities, were analyzed using commercial clinical diagnosis kits according to the manufacturers’ standards and protocols (Jiancheng, Nanjing, China).

### 2.8. Liver Reactive Oxygen Species (ROS) Level 

Small portions of fresh liver tissues were collected and homogenized in ice-cold lysis solutions (10 mM Tris, 150 mM NaCl, 0.1 mM EDTA, 0.5% Triton X 100, pH 7.5). After centrifugation (1000× *g*, 10 min), the supernatant was collected for analysis. The level of intracellular ROS was determined by fluorescence with 2′,7′-dichlorofluorescein (DCF-DA), upon oxidation of non-fluorescent, reduced, DCFH (Dichloro-dihydro-fluorescein) [20]. The fluorescence intensity of the supernatant was measured with a microplate fluorometer (Fluoroskan Ascent, Thermo Fisher Scientific Inc., Waltham, MA, USA) at 488 nm excitation and 535 nm emission. Cellular oxidant levels were expressed as the relative DCF fluorescence per µg of protein (BCA method). 

### 2.9. Statistical Analysis 

The results are presented as the mean ± SEM. The statistical analyses were performed using a one-way ANOVA followed by the least significant different post hoc analyses. For all of the analyses, values of *p* < 0.05 were considered statistically significant.

## 3. Results

### 3.1. The Effects of BG and MLE on Body and Tissue Weights

**Figure 1:**
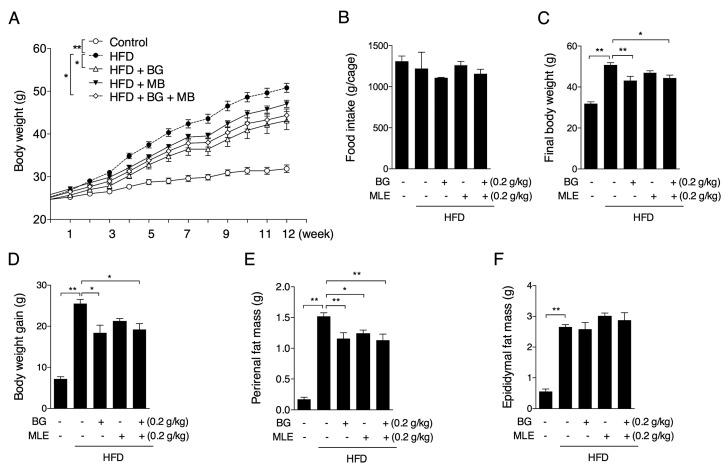
Effects of BG and MLE on body and tissue weights. Body weight curve (**A**); final body weight (**B**); body weight gain (**C**); perirenal fat mass (**D**); and epidydimal fat mass (**E**) in male C57BL/6 mice fed a control diet or a high-fat diet supplemented with BG or/and MLE for 12 weeks. The data are the mean ± SEM, *n* = 10. * *p* < 0.05, ** *p* < 0.01 vs. related control. BG: beta-Glucan; MLE: mulberry leaf extract; HFD: high-fat diet.

The mice were divided into five groups at the initiation of the diet and BG and MLE treatments. The initial average body weight did not differ between the five groups. During the trial period, the body weight of all of the groups increased. The HFD group exhibited a steady increase in body weight from the third week, while the HFD + BG and HFD + BG + MLE groups showed lower increases relative to the HFD group (Figure 1A). The final body weights and body weight gains were significantly higher in the HFD group relative to the control group and were lower in the HFD + BG and HFD + BG + MLE groups (Figure 1B,C). Although the HFD + MLE group trended toward a decreased body weight, no significance difference was observed (Figure 1A–C). A dramatic increase in perirenal fat (Figure 1D) and epidydimal fat mass (Figure 1E) was found in the HFD group compared to the control group. All three supplemented groups showed a reduced perirenal fat mass relative to the HFD group (Figure 1D), while no significant difference was observed in the epidydimal fat mass (Figure 1E).

### 3.2. The Effects of BG and MLE on Serum Lipids 

**Figure 2:**
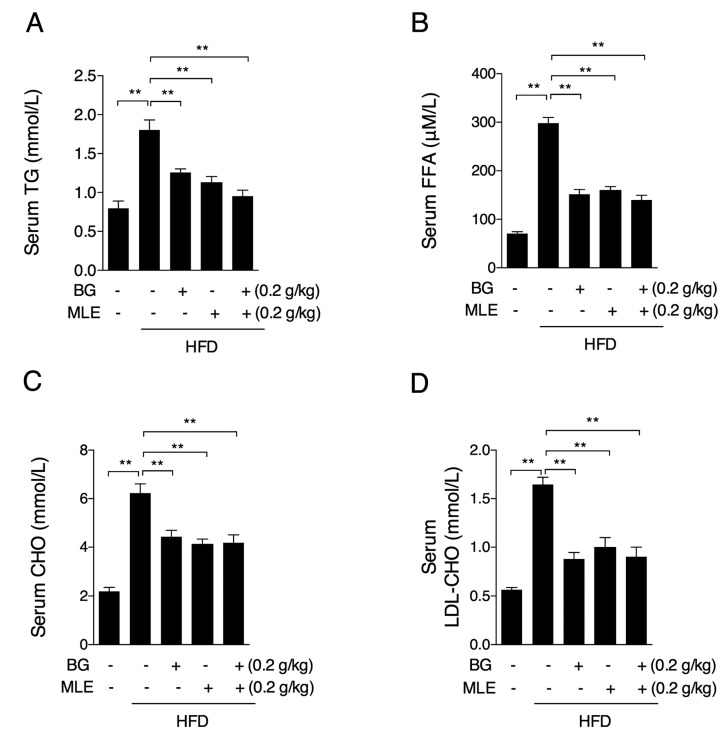
Effects of BG and MLE on serum lipids. Serum TG (**A**); FFA (**B**); CHO (**C**); and LDL-CHO (**D**) in male C57BL/6 mice fed a control diet or a high-fat diet supplemented with BG or/and MLE for 12 weeks. The data are the mean ± SEM, *n* = 10. * *p* < 0.05, ** *p* < 0.01 vs. related control. TG: triglyceride; CHO: cholesterol; LDL-CHO: low density lipoprotein-cholesterol; FFA: free fatty acid.

Hyperlipidemia is one of the major associated factors commonly observed in obesity. In our mice model, we found increased triglyceride levels in the HFD group, as expected (Figure 2A), which were lower in the BG, MLE, and BG + MLE supplement groups (Figure 2A). Consistent changes in free fatty acids (FFA) were also observed (Figure 2B). Total cholesterol also increased in the HFD group and was reduced in the three supplemented groups (Figure 2C). Meanwhile, the HFD group displayed higher HDL-cholesterol levels, which were sufficiently lower in three supplemented groups (Figure 2D). 

### 3.3. The Effects of BG and MLE on Glucose Tolerance 

**Figure 3:**
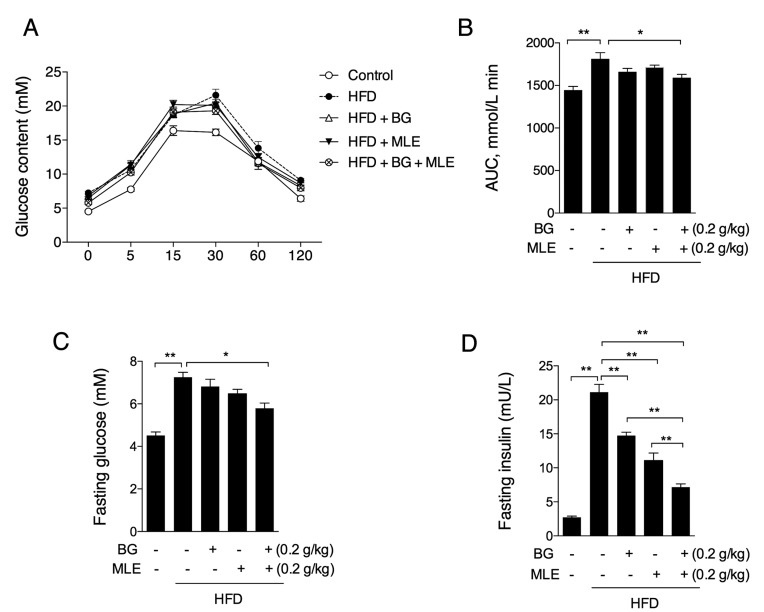
Effects of BG and MLE on insulin sensitivity. OGTT (**A**); fasting serum glucose (**B**); and fasting serum insulin (**C**) in male C57BL/6 mice fed a control diet or a high-fat diet supplemented with BG or/and MLE for 12 weeks. The data are the mean ± SEM, *n* = 10. * *p* < 0.05, ** *p* < 0.01 vs. related control. OGTT: oral glucose tolerance test.

An oral glucose tolerance test was performed during the 11th week of treatment. As shown in Figure 3A, the HFD group displayed significantly lower insulin sensitivity relative to the control group, which was improved in the BG + MLE group, while neither the BG nor the MLE group showed a significant effect for this parameter (Figure 3A). Similar changes were also observed in the mice’s fasting glucose levels, indicating the consistent benefits of BG + MLE on glucose metabolism (Figure 3B). Fasting insulin was largely increased in the HFD group, indicating potential insulin resistance, and all three supplemented groups had significantly lowered insulin levels (Figure 3C). Moreover, it was interesting to observe that the BG + MLE group showed a more significant lowering in fasting insulin compared to either the BG or the MLE group, suggesting a significant synergetic effect of BG + MLE (Figure 3C).

### 3.4. The Effects of BG and MLE on Serum Cytokines

**Figure 4:**
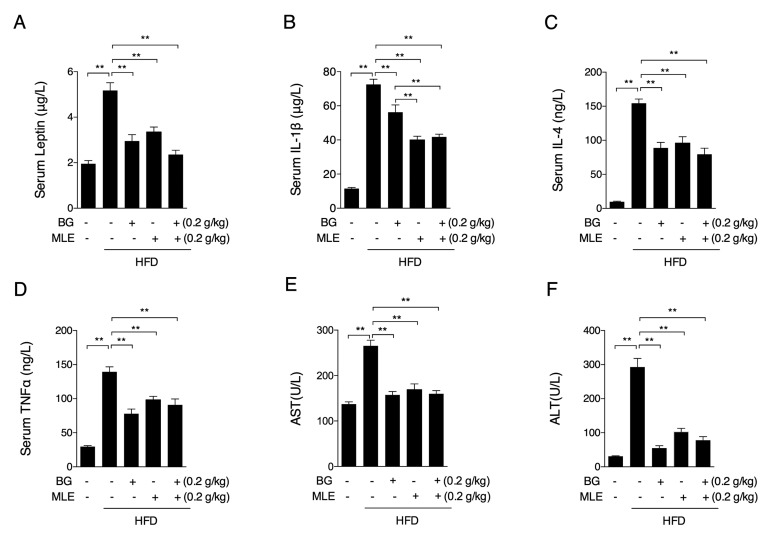
Effects of BG and MLE on serum cytokines. Serum leptin (**A**); IL-1β(**B**); IL-4 (**C**); TNFα (**D**); AST (**E**) and ALT (**F**) levels in male C57BL/6 mice fed a control diet or a high-fat diet supplemented with BG or/and MLE for 12 weeks. The data are the mean ± SEM, *n* = 10. * *p* < 0.05, ** *p* < 0.01 vs. related control.

Long-term feeding results in a reduction in central leptin sensitivity [20]. As expected, the HFD group had significantly higher serum leptin levels, which were lower in all of the three supplemented groups (Figure 4A). Obesity is also associated with elevated levels of proinflammatory cytokines in the circulation and in tissues [21], and thus, we found that IL-1β, IL-4, and TNF-α levels in the serum were increased in the HFD group, and all of the three supplements sufficiently reduced these levels (Figure 4B–D). ALT and AST are tested as serum markers of liver function and are usually abnormal in the obesity condition, and it is well established that nonalcoholic fatty liver disease (NAFLD) is the most frequent chronic liver disease that is strongly linked to obesity [22]. We found increased ALT and AST levels in the HFD group, as expected, and these levels were sufficiently decreased in all of the supplemented groups (Figure 4E,F).

### 3.5. The Effects of BG and MLE on Fatty Liver 

**Figure 5:**
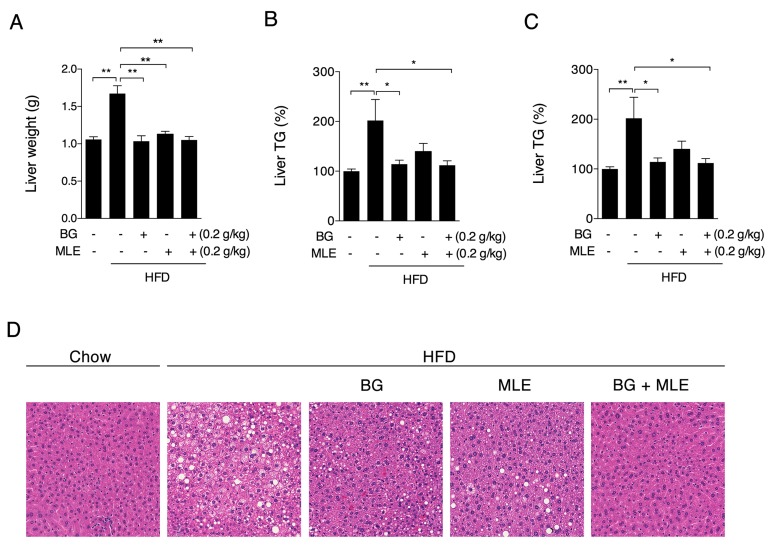
Effects of BG and MLE on liver lipids. Liver weight (**A**); liver TG (**B**); liver CHO (**C**); and HE staining of liver tissue (**D**) in male C57BL/6 mice fed a control diet or a high-fat diet supplemented with BG or/and MLE for 12 weeks. The data are the mean ± SEM, *n* = 10. * *p* < 0.05, ** *p* < 0.01 vs. related control.

Along with abnormalities of liver function markers in the serum, liver weight was significantly higher in HFD groups than the normal diet, which was normalized in all the three supplemented groups (Figure 5A). The lipid analysis showed significantly higher triglycerides and total cholesterol levels in the HFD group than the normal diet, which were decreased by BG, MLE, or BG + MLE supplements (Figure 5B,C). Meanwhile, HE (Hematoxylin and eosin) staining of liver tissues showed consistent changes among the groups (Figure 5D), further indicating the beneficial effects of BG and MLE supplements on fatty liver. 

### 3.6. The Effects of BG and MLE on Liver Oxidative Stress 

**Figure 6:**
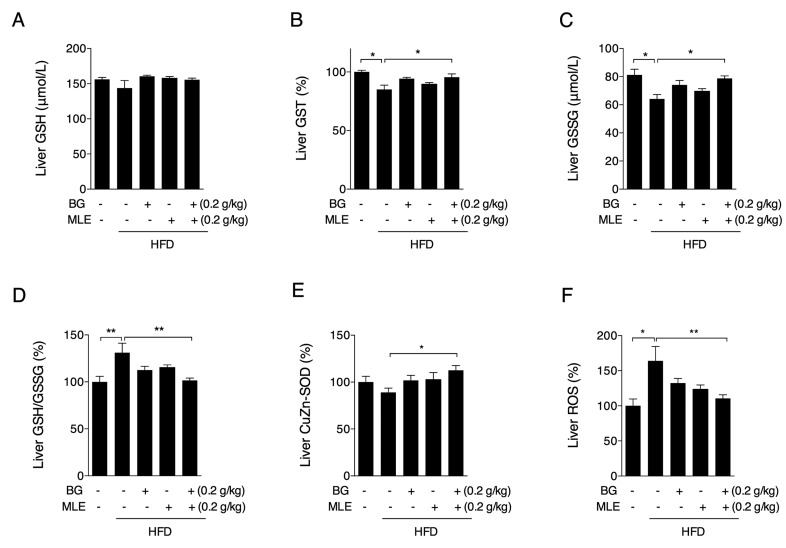
Effects of BG and MLE on liver oxidative stress. Liver glutathione (GSH) (**A**); liver GST (**B**); liver GSSG (**C**); liver GSH/GSSG (**D**); liver Cu-ZnSOD activity (**E**); and liver ROS (**F**) in male C57BL/6 mice fed a control diet or a high-fat diet supplemented with BG or/and MLE for 12 weeks. The data are the mean ± SEM, *n* = 10. * *p* < 0.05, ** *p* < 0.01 vs. related control.

Oxidative stress is one of the known factors that contribute to the development of fatty liver, and we further analyzed the oxidative status of liver tissues. GSH, the most abundant antioxidant in living cells, was not changed among groups (Figure 6A). GST activity was decreased in the HFD group compared to the normal diet, and only BG and MLE showed significant increases (Figure 6B). Consistently, GSSG content was largely decreased in the HFD group, and only BG + MLE showed significant increases (Figure 6C). As a result, the ratio of GSH/GSSG was significantly increased in the HFD group compared to the normal controls, and only the BG + MLE group showed a significant decrease (Figure 6D). Furthermore, we found that the BG + MLE group had significantly higher CuZn-SOD activity than the HFD group (Figure 6E), and BG + MLE was the only group that had a lower ROS content in liver relative to the HFD group (Figure 6F). We thereby concluded that BG and MLE have synergetic beneficial effects on liver oxidative stress.

## 4. Discussion

Obesity is known as a major risk factor for hyperlipidemia, fatty liver and insulin resistance. In the past decades, many studies have investigated potential diets and diet components as a first-line for the intervention and treatment of obesity-associated MS [23]. It was suggested that over 800 plants may have antidiabetic agents [24], while only a small number of these products have received scientific and medical evaluations to assess their efficacy. Among them, BG has been a popular ingredient with widespread attention in the scientific field. Unfortunately, controversial outcomes in clinical studies have occurred that were partially due to obscure mechanisms and large intake doses of BG in animal studies. In the present study, we further demonstrated that a low dose of BG could also benefit obesity and associated metabolic disorders. More importantly, we showed that combination of BG with MLE, another popular dietary component, that is gaining increasing attention in the field of obesity management, has synergetic benefits especially on insulin sensitivity and liver oxidative stress management. 

Previous studies have shown that oral intake of BG at 1 g/kg body weight could efficiently lower body weight, blood glucose and lipids in obese mice [25]. A randomized double-blinded clinical trial reported that BG could increase anti-inflammatory cytokine IL-10, decrease pro-inflammatory cytokine IL-6, and reduce waist circumference in obese subjects [26]. Consistent with previous studies, we showed that administration of BG at 200 mg/kg body weight during the HFD feeding could efficiently lower body weight gain, serum lipids and pro-inflammatory cytokine levels, indicating benefits for obese mice. Unlike BG, MLE has recently started to gain scientific attention, even though it has a long history of medical use in some countries. Previous studies have shown that MLE inhibits preadipocyte differentiation and ameliorates HFD-induced obesity and dyslipidemia [17], and in STZ-induced non-obese diabetic rats, MLE could also inhibit hyperglycaemia and increase insulin secretion, resulting in an improved metabolic status when taken at a dose of 6000 mg/kg [18]. Although MLE, at 200 mg/kg, did not show a significant reduction in body weight gain in the current study, serum lipids and pro-inflammatory cytokines were sufficiently decreased by MLE, showing similar benefits as BG. Despite increasing studies showing the importance of either visceral or subcutaneous fat for the pathogenesis of both insulin resistance and glucose intolerance [27,28,29], an increased adipose mass may not fully contribute to metabolic abnormalities, and there are certain states of increased fat mass with preserved metabolic fitness, which are referred to as a “healthy” expansion of adipose tissue [30]. Therefore, although BG, MLE, or BG + MLE had no significant effect on the epidydimal adipose mass reduction and slightly reduced perirenal fat mass, they sufficiently reduced dyslipidemia and fasting insulin. Moreover, the BG + MLE group showed an even greater reduction in fasting insulin relative to the BG or MLE group, together with significant effects on OGTT and fasting glucose; thus, we concluded that the combination of BG and MLE showed interesting synergetic benefits, improving insulin sensitivity and glucose tolerance. 

It is well established that obesity is accompanied by an increased risk of NAFLD [31], and HFD feeding triggers extra triglyceride and cholesterol accumulation in mice livers, representing an imbalance between the complex interactions of metabolic events [32]. After 12 weeks of HFD feeding, liver weights were significantly higher in the HFD group compared to the control diet group and were accompanied by elevated ALT and AST activities, indicating abnormal liver function. A lipid analysis showed increased triglyceride and total cholesterol levels in the HFD group, which were further confirmed by H & E staining of the liver tissues. Consistent with serum lipid changes, all of the supplemented groups showed significant beneficial changes in liver function and lipid deposits. Among the several risk factors accounting for the development of NAFLD, a redox imbalance is number one, and a “two hit hypothesis” is widely accepted for the pathogenesis of NAFLD [33], where ROS is considered the “second hit,” promoting the progression of NAFLD to a more severe nonalcoholic steatohepatitis (NASH) [34]. Since no inflammation and fibrosis were observed in the HE staining, we assumed that HFD feeding might induce an early stage of NAFLD. Following the analysis of oxidative stress in the liver, we showed that the HFD induced significant increases in the GSH/GSSG ratio and the ROS levels, which were only normalized by the BG + MLE supplement. Taken together, our data indicate that the combination of BG and MLE has a synergetic effect, reducing liver oxidative stress, which may further prevent the progression of NASH and fibrosis.

## 5. Conclusions

In conclusion, the data presented here demonstrate that the administration of BG or MLE at a low dose reduces HFD-induced hyperlipidemia, hyperglycemia, insulin resistance and excessive liver lipid accumulation in mice. More importantly, a combination of BG and MLE showed synergetic benefits, improving insulin sensitivity and decreasing liver oxidative stress. Together, these findings provide further support for the use of a combination of functional food ingredients for managing obesity and associated metabolic disorders.

## Abbreviations

BG	β-glucan
CVD	cardiovascular diseases
HFD	high-fat diet
MLE	mulberry leaf extract
MS	metabolic syndrome
NAFLD	nonalcoholic fatty liver disease
NDA	2,3-naphthalenedicarboxyaldehyde
OGTT	oral glucose tolerance test
ROS	reactive oxygen species
T2DM	type 2 diabetes mellitus
